# Radiographic Evaluation of Mastoid Parameters for Sexual Differentiation in North Indian Population

**DOI:** 10.7759/cureus.16011

**Published:** 2021-06-29

**Authors:** Jigyasa Passey, Suniti Pandey, Nishtha Passey, Rahul Singh, Raveena Singh, Arvind Kumar

**Affiliations:** 1 Anatomy, Hamdard Institute of Medical Sciences and Research, New Delhi, IND; 2 Anatomy, Ganesh Shankar Vidyarthi Memorial Medical College, Kanpur, IND; 3 Physiology, Maulana Azad Medical College, Delhi, IND; 4 Anatomy, Indian Institute of Medical Science and Research, Lucknow, IND; 5 Orthopaedics, Hamdard Institute of Medical Sciences, New Delhi, IND

**Keywords:** morphometry, mastoid triangle, north india, sexual dimorphism, skull radiographs

## Abstract

Introduction: Morphometric differences of several bones form the basis of sexual differentiation. The mastoid triangle has been widely used as a predictor of sexual differentiation. However, the radiographic measurements of the mastoid triangle, which form the clinical alternative of this parameter, have not been studied in the North Indian population. Therefore, we analyzed skull radiographs of live subjects to investigate the effectiveness of the radiographic mastoid triangle in sex determination.

Methods: One hundred skull lateral radiographs (55 male and 45 female) from the digital archives of a tertiary care teaching institute in Northern India were retrospectively analyzed. The following parameters: porion-mastoidale length, mastoidale-asterion length, asterion-porion length, and area of the mastoid triangle were measured. Gender-based differences for these parameters were then calculated for any statistical significance. Further, the low value of Wilks’ lambda, high values of Eigenvalues, and percentage of correct prediction accuracy denoted higher predictive value. Finally, discriminant function analysis was used to predict the relative validity of each measured parameter.

Result: All measured parameters were significantly higher in the male group. The porion-mastoidale length was 32.21±2.15 mm in males and 31.66±3.21 mm in females. The mastoidale-asterion length was 50.00±9.75 mm in males and 49.84±6.97 in females. The asterion-porion length was 44.11±6.82 mm in males and 39.72±5.77 mm in females. The area of the mastoid triangle was 690.74±123.35 mm^2^ in males and 570.57±130.0 mm^2^ in females. The area of the mastoid triangle has the highest relative validity (78%).

Conclusion: Considerable ethnic and racial differences have been observed in the radiographic morphology of the mastoid. The radiographic dimensions of the mastoid are potential predictors of sexual dimorphism. With the use of discriminant function analysis, the current study predicts the effectiveness of the area of the mastoid triangle as a reliable parameter for sexual differentiation in the Northern Indian population.

## Introduction

The mastoid process is a smooth conical prominence projecting from the base of the mastoid portion of the temporal bone. It lies just behind the external acoustic meatus and lateral to the styloid process. The upper extent of the mastoid joins the parietal bone and has the petrosquamous suture extending vertically from it. The posterior border merges with the occipital bone, and the anterior border mingles with the descending part of the squamous area of the temporal bone.

It allows the attachment of muscles such as the occipitofrontalis muscle and certain neck muscles like the sternocleidomastoid and splenius capitis muscles. For anthropologists and forensic experts, the identification of gender is of paramount importance for any skeletal remains. Human anthropologists study sexual dimorphism to understand the evolution of Homosapiens [1]. Bioarchaeologists study sexual dimorphism to recreate the demographic profile of our ancestors. In most cases, the entire skeleton is seldomly obtained. The pelvis is the most sexually dimorphic bone, but its complete specimens are rarely found. Owing to the compact structure and strategic anatomical position, the morphometric study of the mastoid region may be approached for sex differentiation. There exist many qualitative criteria for sexing the skulls, but a quantitative approach can provide more objective and reproducible evidence. Radiographs are commonly advised investigations for skeletal evaluation. The radiographic morphometric differences in mastoid parameters can thus form the basis of sexual differentiation [2]. In the current study, we attempted to evaluate the potential of mastoid triangle measurements for sex determination using electronically archived digital radiographs of skulls.

## Materials and methods

**Figure 1:**
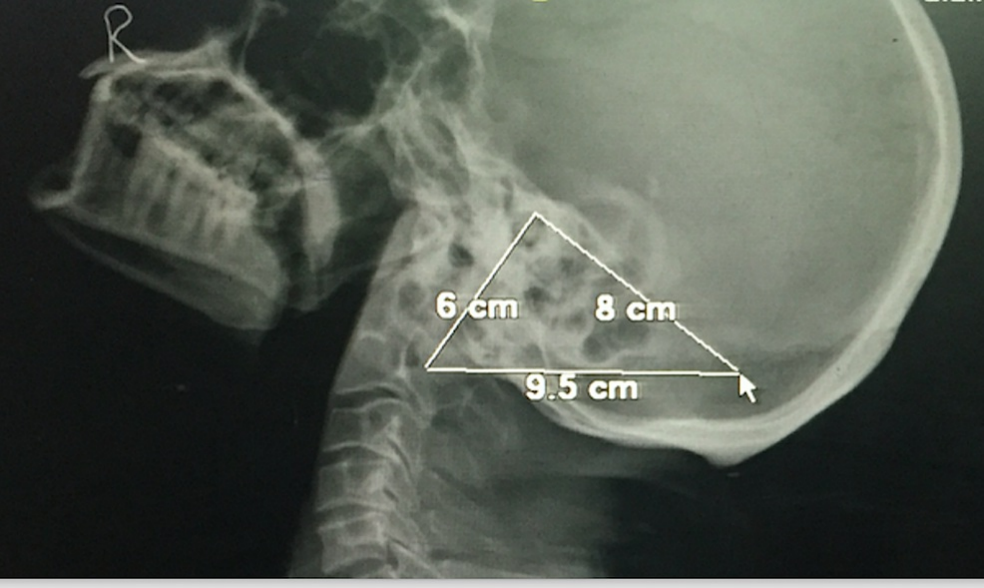
Lateral radiograph of skull showing the mastoid triangle-related measurements

We retrospectively analyzed one hundred lateral radiographs of adult skulls (55 male and 45 female) of live subjects retrieved from the electronic radiograph database of the radiology department of a tertiary care teaching institute in Northern India. Three craniometric points were identified on the skull radiographs: (1) porion which is the uppermost point of the external acoustic meatus; (2) mastoidale which is the inferior most point of the mastoid process; and (3) asterion, which is the meeting point of three posterior skull sutures, i.e., lambdoid, occipitomastoid, and parietomastoid (Figure 1). Only those radiographs with identifiable porion, mastoidale, and asterion were considered for the study. The radiographs with wormian bones and partially fused ectocranial sutures in the asterion’s temporal region were not included in the analysis. The following parameters: porion- mastoidale length, mastoidale-asterion length, asterion-porion length, and area of the mastoid triangle were measured. The mastoid triangle area was calculated using Heron’s formula. The quantitative data were put as mean±standard deviation (SD) and were statistically analyzed to evaluate the differences between male and female groups' measurements. We used an unpaired t-test to compare the individual parametric differences between male and female groups. A p-value of less than 0.05 was considered the marker of statistical significance. A discriminant function analysis was performed on the basis of Wilks' Lambda, Eigenvalues, and the percentage of correct sexual classification of the radiographs by the observer (prediction accuracy) [3]. Interclass correlation of interobserver and intraobserver variation was high for all the measured parameters. Low values of Wilks' lambda, high values of eigenvalue, and higher prediction accuracy denote higher validity of the measured parameter.

## Results

All measured parameters were significantly higher in the male group. The porion-mastoidale length was 32.21±2.15 mm in males and 31.66±3.21 mm in females. The mastoidale-asterion length was 50.00±9.75 mm in males and 49.84±6.97 in females. The asterion-porion length was 44.11±6.82 mm in males and 39.72±5.77 mm in females. The area of the mastoid triangle was 690.74±123.35 mm^2^ in males and 570.57±130.0 mm^2^ in females (Table 1).

**Table 1:** Measurements obtained on the skull lateral radiographs

S.No	Parameter	Male		Female		p-Value	Significance
		Mean	SD	Mean	SD		
1.	Porion-mastoidale (mm)	32.21	2.15	31.66	3.21	0.001	Significant difference
2.	Mastoidale-asterion (mm)	50.00	9.75	49.84	6.97	0.001	Significant difference
3.	Asterion-porion (mm)	44.11	6.82	39.72	5.77	0.002	Significant difference
4.	Area of a mastoid triangle (mm^2^)	690.74	123.35	570.57	130.0	0.001	Significant difference

The area of the mastoid triangle has the lowest Wilks’ lambda, highest Eigenvalue, and the highest sexual prediction accuracy (Table 2). In addition, the discriminant function analysis suggested the highest relative validity of the area of the mastoid triangle.

**Table 2:** Parameter-wise calculation of discriminant functions on the skull lateral radiographs

S.no	Variable	Wilks’ lambda	Eigenvalue	Prediction accuracy %	Rank
						1.	Porion-mastoidale (mm)	0.922	1.851	75.2%	2
2.	Mastoidale-asterion (mm)	0.981	0.588	69%	4
3.	Asterion-porion (mm)	0.976	0.783	70.1%	3
4.	Area of a mastoid triangle (mm^2^)	0.901	1.967	78%	1

## Discussion

The current analysis suggests significant morphometric differences between male and female mastoid parameters in skull radiographs. Sex differentiation is often required in the medicolegal and forensic workup, and radiographs are the easiest readily available skeletal investigations in such scenarios. Our findings suggest that the mastoid triangle measured on skull radiographs is a reliable predictor of gender differentiation in the North Indian population.

The mastoid process is one of the most sexually dimorphic features in the human skull and is often used to identify the sex of skeletons. The sexual dimorphism of the mastoid process has been extensively studied using both the metric and non-metric parameters. Bass et al. [4]. stated that the skull is probably the second-best region of the skeleton to determine the sex. Skull has numerous bony landmarks that can act as potential predictors of sexual dimorphism. Some of the widely studied ones include overall appearance, supraorbital ridges, glabella, zygomatic arches, mastoid processes, external occipital protuberance, mandible, and palate. Hoshi et al. [5] had suggested that when skulls were placed on a flat surface, the male skulls rest on the mastoid processes, while the female skulls rest on the occipital condyles or other portions of the skull. These are, however, the qualitative methods to determine sex. It was also noted that the female skulls retained the infantile type of small mastoid process, whereas males had greater variability. Most previous studies dealt with measuring the mastoid process on dry skulls obtained from exhumed cadavers of known sex, age, and race. The osteometric linear measurements were carried out directly on the skull using calipers. Numerous techniques for assessing variation in the size and shape of the mastoid process have been proposed and implemented in osteological research. However, its complex form still presents difficulties for consistent and practical analysis [6]. A report on evaluating the mastoid triangle for identification of sex was first published by de Paiva and Segre [2] in 2003. The advantages of radiographs are that they are more accurate considering the wear and damage to dry bones and are easier to store as digital records. Farhadian et al. [7] aimed to determine sex in the Iranian population based on measurements of the mastoid process using different data mining algorithms on computed tomography (CT) images. However, radiographs are the frequently ordered and less expensive radiological investigations than CTs, and their analysis is more cost-effective. As discussed in Table 3, the porion-mastoidale, mastoidale-asterion, asterion-porion, and the mastoid triangle area formed measured higher in males than females.

**Table 3:** Morphometric comparison of asterion-porion distance, porion-mastoidale distance, mastoidale-asterion distance, and area of a mastoid triangle on skull lateral radiographs among previous radiological studies As-Po: asterion-porion distance, Ma-As: mastoidale-asterion distance, Po-Ma: porion-mastoidale distance, Area-Ma: area of mastoid triangle, M: male, F: female, R: right, L: left.

S. No	Researcher	Race/region	As-Po (mm)				Po-Ma (mm)				Ma-As (mm)		Area-Ma (mm^2^)	
			M		F		M		F		M	F	M	F
1.	Madadin et al. [8]	Saudi Arabia	44.04±6.65		41.50±6.43		32.68±3.94		29.40±4.72		51.95±9.08	48.42±6.81	692.82±155.82	596.01±145.30
2.	Toneva et al. [9]	Bulgaria	51.97±3.85(R) 51.34±3.80(L)		47.41±3.12(R) 46.99±2.85(L)		33.64±2.94(R) 33.19±2.75(L)		28.55±2.68(R) 28.49±2.79(L)		56.42±4.66 (R) 56.21±4.82 (L)	49.99±4.45 (R) 49.00±4.27 (L)	853.68±104.70 (R) 835.93±103.72 (L)	657.94±85.20 (R) 644.86±82.93 (L)
3.	Jaja et al. [10]	Nigeria	34.3±7.3		33.1±6.3		46.1±11.9		43.0±9.6		47.3±10.4	42.8±8.9	670.3±188.1	590±101.4
4.	Present study	North Indian	44.11±6.82		39.72±5.77		32.21±2.15		31.66±3.21		50.00±9.75	49.84±6.97	690.74±123.35	570.57±130.0

Comparing the previous studies analyzing the predictive accuracy of mastoid parameters, our findings on lateral skull radiographs differed from Madaddin et al. [8], who studied the population of Saudi Arabia and computed the measurements using multidetector CT scans. The authors also analyzed their data with discriminant functional analysis and observed that the Porion mastoidale distance had the highest prediction capacity. Toneva et al. [9] studied the mastoid triangle using 3D models and observed sufficient discriminating power of the mastoid triangle dimensions for sex estimation among Bulgarians (up to 89%). The total mastoid triangle area proved to be the single best sex-discriminating trait which was similar to our results.

According to Jaja et al. [10], the asterion‐mastoidale distance and mastoid triangle area were sexually dimorphic with mean values higher in males compared with females (p = 0.02), and on cross-validation, the triangular area accurately identified 80% of females and 48% of males. Amin et al. [11] revealed that the mastoid process correctly classified the sex in 90.6% of the subjects, and the intermastoidale distance was found to be the best determinant of sex.

Currently, there is a lack of radiographic studies in predicting mastoid-based sexual dimorphism as far as the Indian population is concerned. The current study attempts to fill that lacuna and can help trigger further research in this regard. There were some limitations of this study. First, the study was a preliminary study with a sample size of a hundred, limiting the generalization of findings. Second, the study was based on digitally stored radiographs, and minor errors due to magnifications and orientation of the skull can affect the findings. Third, the findings are less accurate than a CT-based assessment. Lastly, the study does not neutralize the errors due to the mingling of the populations, and the sample might not be entirely representative of the North Indian population. Nevertheless, the study is unique in itself and establishes the radiographic mastoid parameters, especially the mastoid triangle area, as the reliable predictor of sexual dimorphism. The study validates the usage of radiographs in determining the sex using lateral cephalograms and can be potentially helpful for anthropologists and radiologists.

## Conclusions

Considerable ethnic and racial differences have been observed in the radiographic morphology of the mastoid. Radiographic dimensions of the mastoid are potential predictors of sexual morphism. With the use of discriminant function analysis, the current analysis predicts the effectiveness of the area of the mastoid triangle as a reliable parameter for sexual differentiation in the northern Indian population. Further research on a larger scale will help in strengthening this evidence.
